# Metagenome-Scale Metabolic Network Suggests Folate Produced by *Bifidobacterium Longum* Might Contribute to High-Fiber-Diet-Induced Weight Loss in a Prader–Willi Syndrome Child

**DOI:** 10.3390/microorganisms9122493

**Published:** 2021-12-01

**Authors:** Baoyu Xiang, Liping Zhao, Menghui Zhang

**Affiliations:** State Key Laboratory of Microbial Metabolism, and School of Life Sciences & Biotechnology, Shanghai Jiao Tong University, Shanghai 200240, China; bug.baogebbl@sjtu.edu.cn (B.X.); lpzhao@sjtu.edu.cn (L.Z.)

**Keywords:** folate, high-fiber diet, metagenome-scale metabolic network, gut microbiota, obesity

## Abstract

Gut-microbiota-targeted nutrition intervention has achieved success in the management of obesity, but its underlying mechanism still needs extended exploration. An obese Prader–Willi syndrome boy lost 25.8 kg after receiving a high-fiber dietary intervention for 105 days. The fecal microbiome sequencing data taken from the boy on intervention days 0, 15, 30, 45, 60, 75, and 105, along with clinical indexes, were used to construct a metagenome-scale metabolic network. Firstly, the abundances of the microbial strains were obtained by mapping the sequencing reads onto the assembly of gut organisms through use of reconstruction and analysis (AGORA) genomes. The nutritional components of the diet were obtained through the Virtual Metabolic Human database. Then, a community model was simulated using the Microbiome Modeling Toolbox. Finally, the significant Spearman correlations among the metabolites and the clinical indexes were screened and the strains that were producing these metabolites were identified. The high-fiber diet reduced the overall amount of metabolite secretions, but the secretions of folic acid derivatives by *Bifidobacterium longum* strains were increased and were significantly relevant to the observed weight loss. Reduced metabolites might also have directly contributed to the weight loss or indirectly contribute by enhancing leptin and decreasing adiponectin. Metagenome-scale metabolic network technology provides a cost-efficient solution for screening the functional microbial strains and metabolic pathways that are responding to nutrition therapy.

## 1. Introduction

Diverse dietary approaches have been developed in order to curb the worldwide epidemic of obesity [1]. Food intake influences not only the human body but also the microbiota that are colonizing the digestive tract. Estimated to be the same in number as human cells, these large quantities of microbiota rapidly respond to the diet and the metabolites that they produce after food fermentation can impact the host tissues, resulting in beneficial or detrimental effects on human health [2,3]. In recent decades, gut-microbiota-targeted nutrition intervention has received attention for use in the management of obesity and other consequent diseases, such as diabetes, cancer, and hyperlipidemia [4,5,6]. The success of this treatment has mainly been attributed to its modulation of microbial dysbiosis, which is usually deduced from partial evidence [6]; for instance, the observed shift in the microbial structure after intervention, functional demonstration of a few selected strains, etc. Generally, these approaches cannot provide enough detailed information in order to systematically explain the underlying mechanism [7].

Determining the metabolites that are produced by the gut microbiota is important in understanding how the gut microbiota changes in response to the diet and how the microbiota influence the host afterward. However, this is challenging since current experimental technology is not able to fully profile the metabolites, due to the complexity of the gut microbiota and the interactions among the microbiota communities [8]. With the development of sequencing technology and the quickly increasing knowledge about microbiota, inferring the function of gut microbiota through metagenome data has become feasible. A widely used approach is to infer the metabolites and functional information by mapping microbial sequencing reads or contigs to functional databases. However, the results from these mappings lack precision since the presence of a particular gene does not guarantee the expression of a functional enzyme [9]. Metabolic network simulation provides an alternative method that can be used to simulate metabolite production, based on metagenome data and the previously verified metabolic knowledge of the microbes [10]. A metabolic network contains nodes indicating metabolites and edges indicating biological processes such as conversions, uptake, and secretion [8]. Compartments are used to simulate how different cells metabolically interact with each other [8]. Several methods are available to complete metabolic network simulation, such as flux balance analysis (FBA) and dynamic FBA (DFBA). FBA works at a steady-state, whereas DFBA works for dynamic changes so it can only be used in small communities, due to the dramatically increased time and costs of simulation [11,12,13,14]. In FBA, the reactions in the network are represented by a set of linear equations, constraints are used to limit the flow of metabolites through the network, and the distribution of metabolic fluxes in the metabolic network is calculated for a given objective function. FBA is usually used to estimate the metabolic viability of a microbial community under different conditions, as well as the effect of adding new species to a bacterial community on host health [15]. 

Several software programs have been developed to simulate networks, such as OptCom [16], BacArena [17], MiMoSa [18], FLYCOP [19], MICOM [9], Metage2Metabo [20], and The Microbiome Modeling Toolbox [21]. The Microbiome Modeling Toolbox is based on the constraint-based reconstruction and analysis (COBRA) approach, which provides a molecular mechanistic framework for the integrative analysis of experimental data and quantitative prediction of physiochemically and biochemically feasible phenotypic states [22]. It can model microbial communities using microbial genome-scale metabolic reconstruction data by using flux variability analysis (FVA) [21,23]. Genomic-scale metabolic models (GEMs), also known as genomic-scale metabolic reconstructions (GENREs), are essential for metabolic network simulation. Several tools are available for constructing GEMs [24]. As not all genes of an organism are active, the constraints of these automatically generated GEMs should be manually refined. Some databases such as BiGG [25], KBase [26], and CarveMe [27] provide GEMs. AGORA is a recently widely used, semi-automated database that contains 818 human gut GEMs (we used 773 GEMs in this research, v.1.03) and their gene sequences [28]. Since a wider variety of organisms is included in the database, the GEMs can be more generally constructed, enabling the use of these GEMs in different ways. Using the Microbiome Modeling Toolbox and AGORA, researchers found the correlation between microbes, metabolites, and host diseases such as *Clostridioides difficile* infection and inflammatory bowel disease [29,30,31,32]. 

In our previous trial, we found that a dietary intervention can reshape the gut microbiota and recover host health [3,33]. Diets that are rich in undigestible (but fermentable) carbohydrates could significantly promote beneficial bacteria and reduce toxin producers, which might contribute to the alleviation of metabolic deterioration in both simply and genetically obese children, regardless of the primary forces driving the obesity [33]. From the gut microbial analysis of the composition, single nucleotide polymorphisms (SNPs) were found to occur, with virulence factors (VFs) being carried, indicating that the dietary intervention significantly changed the gut microbiota structure, genes, and genetic properties [33,34,35,36]. Since the metabolites that are produced by the gut microbiota play an important role in host–microbiota interactions, in this study we investigated how the metabolites change during a dietary intervention and how these metabolites influence host health conditions. 

An obese child with Prader–Willi syndrome (PWS) was selected as the representative case in this study. This child’s body weight reduced from 140 to 114 kg after 105 days’ dietary intervention and both the plasma glucose and lipid homeostasis improved to within the normal ranges [36]. Two systemic inflammation markers, C-reactive protein (CRP) and serum amyloid A protein (SAA), also decreased. His adiponectin increased from 2.17 to 5.39 μg/mL and his leptin decreased from 63.8 to 34.5 ng/mL. In addition, the amount of lipopolysaccharide-binding protein (LBP), a surrogate marker for the bacterial antigen load in the blood [37], decreased. The focus of this study was to screen for the functional microbial strains and metabolic pathways that might be important for weight loss during a dietary intervention. We first built individual metabolic networks for each sampling time point using the fecal metagenomic data and the nutrition information of the given diet. Then, we focused on the systematic obesity-relevant metabolic networks containing metabolites, microbiota, and clinical parameters. Finally, key metabolites and their microbial producers were screened out.

## 2. Materials and Methods

### 2.1. Clinical Investigation

All the data from the PWS obese child (GD02, 14 years old, boy) that were used in this study were obtained from a hospital-based intervention, which was performed at Guangdong Women and Children Hospital in Guangzhou, China. In this intervention, 38 children in total (17 with PWS and 21 with simple obesity, aged from 3–16 years old) had received a high-fiber dietary therapy in order to alleviate their obesity. The diet was prepared in the form of ready-to-eat foods with three formulas (see the first column in Table 1), containing whole grains, traditional Chinese medicinal foods, and prebiotics (manufactured by Perfect (Zhongshan, China) Co., Ltd.) [38]. Additionally, the children were provided with appropriate amounts of vegetables, fruits, and nuts according to a dietician’s advice. The children were not asked to participate in any exercise program and there was no limit to the amount of Formula 1 that they ate, but the provision of the other two formulas was controlled. The intervention duration for the group of simple obesity children was 30 days and for the PWS children it was 90 days; except in the case of GD02, who had stayed in the hospital for a further 285 days since the point at which his BMI was measured at 49.47 kg/m^2^ on day 90 and he was willing to continue the intervention. As GD02’s compliance was good and his case had longer time point measurements available that might be better suited for model construction, particularly with high-throughput data, we chose this child as the representative in this metagenome-scale metabolic study. GD02’s anthropometric and clinical indices (BMI, leptin, oral glucose tolerance test (OGTT), insulin AUC, adiponectin, FFA, TNF-α, fasting plasma glycemia (FPG), and total cholesterol) were measured at the hospital on the intervention days 0, 15, 30, 45, 60, 75, and 105, and fecal microbiome sequencing data that were collected at these corresponding sampling times were used [33].

The hospital-based intervention was performed under the approval of the Ethics Committee of the School of Life Sciences and Biotechnology, Shanghai Jiao Tong University (No.2012-016). The clinical trial was registered with the Chinese Clinical Trial Registry (ChiCTR-ONC-12002646). Written informed consent was obtained from the guardians of all the participants.

### 2.2. Metagenomic Sequencing and Data Preprocessing

The fecal samples that were taken from GD02 on intervention days 0, 15, 30, 45, 60, 75, and 105 were frozen on dry ice immediately after collection and stored at −80 °C until further analysis was undertaken. The DNA was extracted as previously described [39] and metagenomic sequencing was performed using an Illumina HiSeq 2000 platform at Genergy Biotechnology (Shanghai, China) Co., Ltd. The DNA library preparation, cluster generation, template hybridization, isothermal amplification, linearization, blocking, and denaturing and hybridization of the sequencing primers were all performed according to the workflow that was indicated by the manufacturer. Paired-end reads with 151 bp in both the forward and reverse directions were obtained. These original sequencing data can be accessed at the NCBI SRA database with accession number SRP045211.

Trimmomatic (v.0.39) was used to trim the adapters and to control the quality of the sequencing reads, including (i) trimming the reads from 3’ and 5’ until the first nucleotide with a quality threshold of 6 was reached, (ii) removing the reads that were scanned in a 4-base sliding window with an average quality per base below 20, and (iii) removing the read pairs that were less than 60 bases long. The remaining reads that were able to be aligned to the human genome (*Homo sapiens*, UCSC hg19) with Bowtie2 (v.2.3.5.1) were also removed. On average, 25.2 × 10^6^ ± 3.98 × 10^6^ (mean ± SD) paired-end high-quality reads for each sample were retained and used for the downstream analysis.

### 2.3. Quantitative Calculation of Diet Component Intakes 

The details regarding the type and quantity of the nutrition components that were contained in each food that was included in the diet were mainly obtained through the Virtual Metabolic Human (VMH) database. For the foods that were not contained in the VMH database, such as adlay, the nutrition components were searched in the literature [40,41] and manually added as a VMH nutrition type. The total intake quantity of each dietary component per day was summed according to the food intake records (Table 1). VMH’s “design a diet” function [42] was used to integrate all the dietary components that were consumed and to transform them into quantitative fluxes of metabolites in preparation for metabolic network construction (see Tables S1–S3 in Additional File S1).

### 2.4. Construction of Metagenome-Scale Metabolic Network

For each sample that was taken at the different time points, an individual metagenome-scale metabolic network was constructed using the quantitatively calculated fluxes and the high-quality sequencing reads that were obtained previously. First, the high-quality reads of a sample were mapped onto the reference set of 773 AGORA genomes with CoverM (v.0.6.1) [43] in order to obtain the abundances of the microbes. Then, in the MATLAB environment (v.R2019b, MathWorks (Natick, MA 01760 USA), Inc.) using Gurobi as the linear and quadratic programming solver, the metabolic simulations were completed with functions that were implemented in the Microbiome Modeling Toolbox [21]. Briefly, the microbial community models for each sample were constructed with AGORA (v.1.03), based on their abundances. Only the microbes with a relative level of abundance above 0.1% were used in the metabolite simulation. Each community model was then simulated under a given diet using FVA to simulate the maximal and minimal abundance of each metabolite’s uptake and secretion. The net uptake was obtained by summing the maximal uptake and minimal secretion, whereas the net secretion was calculated as the sum of maximal secretion and minimal uptake. Finally, for each metabolite at each sampling time point, the contributions from the corresponding microbes were identified and calculated. In this section, unless otherwise stated, for all of the calculations we used the default parameters that were set by the software. 

### 2.5. Statistical Analysis

The Spearman correlations among the metabolites, microbes, and host’s clinical parameters were calculated in R (v.4.0.3) using the corr.test function. The *p*-value was post-adjusted by using the FDR method [44].

### 2.6. Data Visualization

The contributions of the microbial species to the amounts of the metabolites were plotted using the online implementation of Circos [45,46]. Cytoscape software (v.3.8.2) was used in order to construct a correlation network among the metabolites, microbes and host’s clinical parameters. All of the other data were visualized in R (v.4.0.3), including the following packages: corrplot, ggplot2, pheatmap, psych, reshape2, dplyr, NbClust, VennDiagram, and scico.

## 3. Results

### 3.1. Persistent Variation in Gut Microbiota during Dietary Intervention 

**Figure 1:**
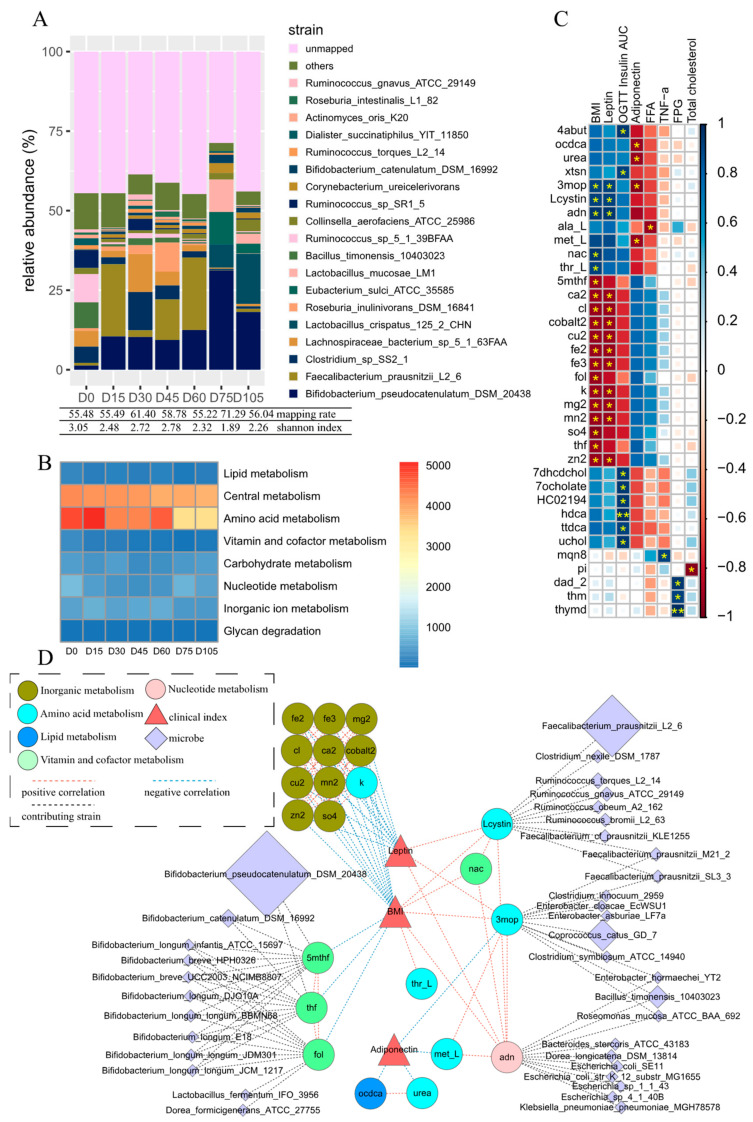
Results of metagenome-scale metabolic network simulation. (**A**) The relative abundance of gut strains at different dietary intervention timepoints. Data were obtained by mapping high-quality reads to the AGORA reference genomes. For the mapped strains, only the most abundant 19 strains are shown and the remaining strains are summed and labeled as “others”. The unmapped reads are assigned as “unmapped”. (**B**) The summed abundances (mmol/day) of potential metabolite secretion in metabolism subsystems at different dietary intervention timepoints. (**C**) Correlations among host clinical parameters and the simulated metabolites of gut microbiota. Each row represents a metabolite and each column represents a clinical parameter. Correlations were calculated with Spearman correlation and post-adjusted with FDR. ‘*’: R > 0.7, *p*-value < 0.1, ‘**’: R > 0.7, *p*-value < 0.05. color of each rectangle in each cell represents *p-*value while size represent R. (**D**) Network diagram among the key metabolites and their contributing strains related to the obese child’s BMI, leptin, and adiponectin. The BMI, leptin and adiponectin are expressed in red triangles, the correlated metabolites are expressed in ellipses and the strains are expressed in diamonds. Different metabolisms are distinguished with colors. Only correlations with R > 0.7 and *p*-value < 0.1 are shown. Red lines indicate positive correlation while blue lines represent negative correlation. Metabolites and their producing strains are linked with black lines. FPG: Fasting Glycaemia, OGTT: oral glucose tolerance test, abbreviation for metabolites is from VMH database, 3mop: 3-methyl-2-oxopentanoate, 5mthf: 5-Methyltetrahydrofolate, adn: Adenosine, ca2: calcium, cl: Chloride, cobalt2: Co^2+^, cu2: Cu^2+^, fe2: Fe^2+^, fe3: Fe^3+^, fol: folate, k: potassium, Lcystin: L-cystine, met_L: L-methionine, mg2: magnesium, mn2: Mn^2+^, nac: nicotinate, ocdca: octadecenoate, so4: sulfate, thf: 5,6,7,8-Tetrahydrofolate, thr_L: L-threonine, urea: Urea, zn2: Zinc.

The relative abundances of the gut microbiota at the different dietary intervention timepoints were obtained by mapping the high-quality reads to the AGORA reference genomes [28]. The mapping rates of these reads at the strain level ranged from 55.5% to 71.3%, without an obvious link to the alpha diversity of the fecal microbiota samples (Figure 1A). The number of the mapped strains was 154 and the minimal abundance of these mapped strains was 0.0039. When compared with intervention day 0, the Shannon indices taken after intervention had all decreased, which indicated that the implemented high-fiber diet had reduced the alpha diversity of the community. Although the applied diet components remained relatively stable during the intervention, the structure of the gut microbiota persistently varied with time. The dominant strains that were increased by the diet mainly included *Bifidobacterium pseudocatenulatum DSM 20438*, *Faecalibacterium prausnitzii L2_6*, and *Clostridium* sp. *SS2_1*. These strains did not continuously increase, but instead fluctuated after being raised, which indicated a continuous balance among the community members.

### 3.2. Metabolites Simulated from the Reconstructed Microbial Metabolic Network

For each of the samples from the different timepoints, a metagenomic-scale metabolic network was individually and quantitively constructed using the calculated diet and the known abundances of the mapped strains. A total of 88 strains had a relative abundance above 0.1% and they were involved in 2908 reactions, resulting in 338 metabolites in total. These metabolites can be secreted/co-secreted by single or multiple microbes and many of them can then be taken up by other microbes. Only 185 of the 338 metabolites were overall net secreted; of which, 64 can both be taken up and secreted, while the remaining 121 can only be secreted. In addition, another 11 metabolites can only be taken up by the microbiome. The net secreted metabolites mainly belonged to the amino acid metabolism and central metabolism (Figure 1B). In comparison to the beginning of the intervention, nearly all secretions in the subsystems decreased, particularly those in the vitamin and cofactor metabolism subsystems, which decreased from 262.3 on day 0 to 63.6 on day 105. These systematic decreases might be partially due to the reduced intake of the main food (Formula 1) during the intervention. An explanation for this hypothesis is that, although the provided indigestible (but fermentable) carbohydrates were added, the total carbohydrate metabolism did not change much. Furthermore, different changing modes within the same metabolism subsystem were observed; for instance, though the overall vitamin and cofactor metabolism subsystem reduced, the internal secretion of folate increased.

### 3.3. Key Metabolites Associated with Obesity-Relevant Clinical Parameters 

The obese child who was tracked in this study lost weight, progressing from 140.1 kg on day 0 to 114.3 kg on day 105 after the fiber-rich dietary intervention. As the focus of this study was to construct a systematic obesity-relevant metabolic network, we first checked the child’s clinical and biological parameters (leptin, OGTT insulin AUC, adiponectin, FFA, TNF-α, FPG, and total cholesterol) that are thought to be related to a change in BMI. Only leptin and adiponectin showed significant correlations with the BMI. The former was positive (r = 0.89, *p* = 0.0068, without FDR adjustment) and the latter was negative (r = −0.86, *p* = 0.013, without FDR adjustment) (Figure 1C). Notably, the decreased leptin and the increased adiponectin occurred not only in this boy, but also in all of the other obese children who received the same dietary intervention [33]. Furthermore, the leptin/adiponectin ratio showed more significant correlations with BMI (r = 0.93, *p* = 0.0025, with FDR adjustment). These results are consistent with previous reports stating that leptin and adiponectin levels are obesity-relevant [47,48]. 

From the reconstructed microbial metabolic network covering all microbial metabolites and host measurements, 36 metabolites in total were related to at least one clinical measurement of the host (Spearman correlation r > 0.7 and FDR post-adjusted *p* < 0.1), and 22 of these were linked to BMI, leptin, and adiponectin (Figure 1C). Among these 22 metabolites, 4 (fol: folate, thf: 5,6,7,8-Tetrahydrofolate, 5mthf: 5-Methyltetrahydrofolate, and nac: nicotinate) were from the vitamin and cofactor metabolism, 10 (fe2: Fe^2+^, fe3: Fe^3+^, mg2: magnesium, cl: Chloride, ca2: calcium, cobalt2: Co^2+^, cu2: Cu^2+^, mn2: Mn^2+^, zn2: Zinc, and so4: sulfate) were from inorganic metabolites, and 6 (k: potassium, Lcystin: L-cystine, 3mop: 3-methyl-2-oxopentanoate, thr_L: L-threonine, met_L: L-methionine, and urea: Urea) were from the amino acid metabolism. For the remaining two metabolites, octadecenoate (ocdca) is derived from the lipid metabolism and Adenosine (adn) is derived from the nucleotide metabolism.

As the metabolic network was constructed without prior hypothesis or bias, it was an interesting surprise to find that three folic acid derivatives (fol, thf, and 5mthf) from the vitamin and cofactor metabolism were directly negatively correlated with the BMI. Folates are essential cofactors in the metabolic pathways that facilitate biological methylation and nucleotide synthesis and, therefore, they are known to have widespread effects on health and diseases. A literature search indicated that obesity was positively correlated with red blood cell folate, but negatively correlated with serum folate and folate intake [49]. In addition, a low folate intake and low serum levels were found to be associated with a higher BMI and greater abdominal fat accumulation [50]. Hence, we suggest that the negative correlation between folate and BMI shown here could be supported by this evidence to a certain degree. Another metabolite that is also derived from the vitamin and cofactor metabolism, nac, was positively correlated with BMI and we were not able to find obesity-relevant evidence.

The inorganic metabolites, such as ca2 and cu2, were also negatively correlated with BMI and leptin. Some research has indicated that there is a negative correlation of Fe [51] and ca2 [52] to BMI, but a positive correlation between BMI and Zinc has also been found [53]. Until now, how these inorganic metabolites impact on the BMI still remains elusive and further exploration is needed. Notably, k was classified into the amino acids metabolism as based on previous research [31], but in our simulation, it acted more like a member of the group of inorganic metabolites.

The five metabolites from the amino acids metabolism (3mop, Lcystin, thr_L, met_L, and urea) were all either positively correlated with BMI and leptin, or negatively correlated with adiponectin (except for k, which was positively correlated). Among these, 3-methyl-2-oxovaleric acid (3mop), an abnormal metabolite that arises from the incomplete breakdown of branched-chain amino acids, had the most connections; including BMI, leptin, adiponectin, and metabolites such as Lcystin, met_L, and adn, indicating its importance in the regulation of BMI. The detrimental role of 3mop has been reported: it can cause damage to nerve cells and nerve tissues; induce acidosis, which has multiple adverse effects on many organ systems; and may cause adverse health effects at chronically high levels [54]. L-cystine (Lcystin), an oxidized dimeric form of cysteine, also had multiple links to BMI, leptin, 3mop, and nac, suggesting its direct and indirect adverse impacts on BMI. Cystine has been found in high concentrations in the cells of the immune system, skeleton, connective tissues, skin, digestive enzymes, and in hair. Reports have indicated that cystine is associated with risk factors for cardiovascular disease (CVD) including ageing, smoking, obesity, and alcohol abuse [55]. Methionine (Met_L) and urea (the principal product of protein catabolism), along with octadecanoic acid (ocdca) from the lipid metabolism, were negatively correlated with adiponectin. Few reports have linked these metabolites with obesity. L-threonine (Thr_L) was positively correlated with BMI, a finding which also lacks further evidence to prove this correlation.

Similar to 3mop, adenosine (adn) from the nucleotide metabolism was also positively correlated with BMI and leptin and negatively correlated with adiponectin. Due to the existence of multiple receptors, adn performs a broad range of activities in organisms. The concentration of adn affects the functions of the receptors and proteins that evolved in adn synthesis, degradation, and transport. All adenosine receptors were reported to be involved in glucose homeostasis, inflammation, adipogenesis, insulin resistance, and thermogenesis, indicating that adenosine participates in the process of obesity [56]. It was reported that the level of nucleoside adenosine is higher in individuals with obesity and that the specific activation of adenosine receptors could aid in the prevention of obesity [56,57]. Positively linked with the detrimental 3mop and negatively with met_L, adn also behaved as a negative metabolite in our study.

### 3.4. Microbes Contributing to Key Obesity-Related Metabolites

**Figure 2:**
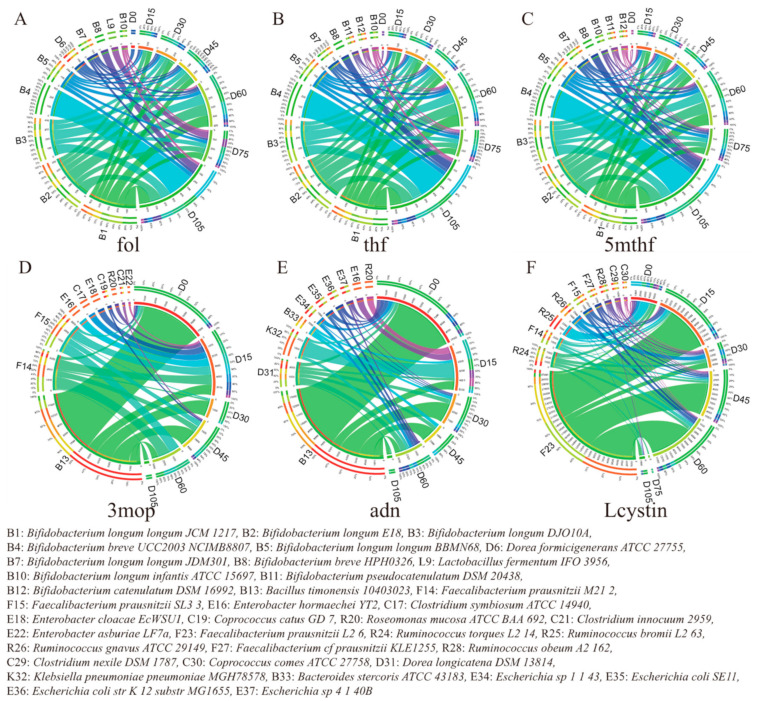
The contribution of strains to metabolites (**A**) fol (**B**) thf (**C**) 5mthf (**D**) 3mop (**E**) adn, and (**F**) Lcystin at different dietary intervention timepoints. Each circle represents a metabolite with its top 10 contributing strains (left part of the circle). The right part of each circle represents the 7 different timepoints.

Based on the reconstructed microbial metabolic network, the strain contributions to each metabolite were calculated. A scheme network indicating the correlations among the key metabolites, the strain contributors to the key metabolites, and the three obesity-relevant clinical parameters (BMI, leptin, and adiponectin) was then drawn, as shown in Figure 1D. As shown, the three folates, folate (fol), tetrahydrofolate (thf), and 5 methyltetrahydrofolic acid (5mthf), that negatively correlated with BMI are mainly produced by strains of *Bifidobacterium longum*, a category of well-established and multifunctional probiotics (Figure 1D). These strains increased during the dietary intervention. Notably, the strains that were contributing to folate varied with time (Figure 2). On day 0, only a few microbes in the gut could produce folate and the other microbes which had the ability to produce folate were considered not to contribute due to their abundances being below the cutoff. During the intervention, the abundance of *Bifidobacterium longum* increased and more microbes began to contribute folate, resulting in increased secretions. On day 105, more than 30% of the folate was produced by *Bifidobacterium breve UCC2003*.

Nearly every microbiome was able to produce the inorganic metabolites that were also negatively correlated with BMI and leptin, but their total abundances were much lower than those of the other metabolites.

For the rest of the key metabolites that positively correlated with BMI and leptin or negatively correlated with adiponectin, 3mop was mainly produced by *Bacillus timonensis 10403023, Faecalibacterium prausnitzii M21_2,* and *Faecalibacterium prausnitzii SL3_3.* The dietary intervention reduced the abundance of these bacteria and thus reduced the abundance of 3mop, which might therefore have lessened the influences on the host. Lcystin was mainly produced by *Faecalibacterium prausnitzii L2_6*, *Ruminococcus torques L2_14*, and *Faecalibacterium prausnitzii M21_2.* In our simulation, the decreased secretion of Lcystin was mainly caused by the reduced abundance of *Faecalibacterium prausnitzii L2_6* and *Ruminococcus torques L2_14*. The metabolite adn was mainly produced by *Bacillus timonensis 10403023, Dorea longicatena DSM 13814,* and *Klebsiella pneumoniae pneumoniae MGH78578.* Particularly, on day 0, *Bacillus timonensis 10403023* produced more than 80% of the 3mop and 65% of the adn and the reduction in this strain was primarily responsible for the decrease in these two metabolites in the following intervention days.

## 4. Discussion

In this case study, a metagenome-scale metabolic network was reconstructed with a focus on screening the microbial metabolic pathways that were involved in reducing the body weight of an obese PWS child under an effective high-fiber dietary intervention. This systematic investigation suggested that, in this child, the weight loss effect might have mainly been achieved through increasing folic acid derivatives’ (fol, thf, and 5mthf) secretion via the vitamin and cofactor metabolism by *Bifidobacterium longum* strains, as well as reducing the metabolites (3mop, Lcystin, and adn) from the amino acid metabolism or nucleotide metabolism that were produced by multiple microbes such as *Bacillus timonensis 10403023* and *Faecalibacterium prausnitzii* strains. These reduced metabolites might also be related to the increase in leptin and the decrease in adiponectin, further contributing to the weight loss of the child. In addition, the overall reduced microbial metabolisms in the gut under the high-fiber diet might have contributed to the weight loss that was observed.

Compared with the association studies that are popularly used in gut microbiota research, the application of a metagenome-scale metabolic network reconstruction displays an advantage in linking the metabolites with their microbial contributors based on verified biological and biochemical knowledge, which helps to elucidate underlying biological mechanisms in detail, and, more importantly, to identify the key functional microbes. Our previous study that was based on the co-abundance gene groups (CAG) analysis [33] and whole-genome comparative analysis of isolated strains [36] showed that obesity might be mainly alleviated by *Bifidobacterium pseudocatenulatum* strains, which were predominantly increased under the high-fiber environment due to their outperforming ability in using complex carbohydrates and their SCFA-producing abilities. Although *Bifidobacterium longum* was positively linked to *Bifidobacterium pseudocatenulatum* in the present study, it was not considered to be the most important functional microbe, due to its relatively low abundance. The relative abundance of *Bifidobacterium longum* was below 3% in our intervention process, while that of *Bifidobacterium pseudocatenulatum* was in the range of 20–40%. However, our findings suggest that obesity might be heavily associated with the microbial production of folate and decreases in 3mop, Lystin, and adn. The folate was mainly produced by *Bifidobacterium longum* strains, instead of *Bifidobacterium pseudocatenulatum*. In addition, among the top ten most abundant bacteria in this study, only the most abundant strain, *Bifidobacterium pseudocatenulatum DSM 20438*, could produce little folate and its production was not correlated with BMI. Due to the complexity of the human gut microbiome, non-infectious disease studies tend to pay more attention to microbes with higher abundance. Our results imply that the bacteria of medium abundance should not be ignored. The identification of the importance of *Bifidobacterium longum* in obesity treatment through folate production in this study is of note. As a beneficial probiotic, *Bifidobacterium longum*’s functioning has been extensively studied. The effects of folate production by *Bifidobacteria* on human health indicate its potential as a probiotic [58] and different strains are known to produce different amounts of folate [59]. Folate was reported to be important for reducing obesity; acquired folate deficiency is quite common and is associated with poor diet and malabsorption, alcohol consumption, obesity, and kidney failure [60]. Though we could not provide experiment verification due to inapplicability, this evidence supports our findings that high-fiber-induced folate secretion by *Bifidobacterium longum* strains is important in this kind of nutrition therapy that is used for treating obesity.

Our metabolites simulation showed that the mathematical relationship analysis between the concentration of metabolites and the abundance of microbes was not always trustworthy. For instance, some metabolites might be well-correlated with some microbes without real producing ability and some microbes may not be related to their metabolites when they are only a member within the relevant reaction chains. In real ecology, these situations are hard to identify. Metabolic network simulation is a suitable technology for use in assisting us in identifying the real mechanism and screening for potential functional microbes.

Some limitations were found in this study. The metagenome-scale metabolic network was restricted to the limited number of strains (773) that have been listed by the AGORA database and the manually curated metabolic models based on prior knowledge. In our case, the mapping rate of our high-quality reads to the AGORA database ranged from 55.5% to 71.3%, so many strains might not have been included in the network. Furthermore, some functions were also excluded from our calculation due to the incompleteness of the models. Additionally, similar to some recent works [29,31], we did not add the human metabolic model to the metabolic network. Although there are some studies of human metabolic models [61], it is still difficult to merge human metabolic models with microbiota community metabolic networks. Some efforts to this end are underway. Jun et al. tried another method in order to estimate the spatiotemporal resolution of the microbial variations in species-level abundance profiles across site-specific colon regions and in feces [62], but many fewer species were employed in the current format of that framework than in AGORA. Finding functional dietary components is important to improve therapeutic design. However, the present modeling system is not able to perform nutritional source-tracing due to the multiple and complex pathways of food digestion. Tracible and quantitative metabolite modeling needs to be developed in the future. Nevertheless, with the rapid development in the field of microbiology, we believe that knowledge of microbial strains and their functions will increase quickly, which will improve the integrity and the precision of metabolic networks.

As it is becoming clear that the gut microbiota at the strain-level is specific to the individual and that strains from the same species might vary widely in their functions and in response to the same diet, metagenome-scale metabolic network technology has a unique advantage in grasping the individual’s overall metabolisms and in obtaining generalized information from the population. We think that the methodology that was used in this study provides a powerful aid in guiding or evaluating personalized nutrition.

## 5. Conclusions

In the current study, we reconstructed a metagenome-scale metabolic network screening the microbial metabolic pathways that are involved in reducing the body weight of an obese PWS child under an effective high-fiber dietary intervention. This systematic investigation suggested that the weight loss effect in this child might have been predominantly achieved through the increasing folic acid derivatives (fol, thf, and 5mthf) that were secreted via the vitamin and cofactor metabolism by *Bifidobacterium longum* strains, and the reduction of the number of metabolites (3mop, Lcystin, and adn) from the amino acid metabolism or nucleotide metabolism, produced by multiple microbes such as *Bacillus timonensis 10403023* and *Faecalibacterium prausnitzii* strains. This study’s findings show that metagenome-scale metabolic network technology provides a cost-efficient solution for screening for the functional microbial strains and metabolic pathways that are responding to a nutrition therapy.

## Figures and Tables

**Table 1:** Food intakes on different sampling days (g/day).

Food Name	Day 0, 15, 30	Day 45, 60	Day 75, 105
**Formula 1**			
Adlay	222	128	123
Hyacinth beans	111	64.3	61.8
Buckwheat	111	64.3	61.8
Oats	166	96.5	92.7
Yam	111	64.3	61.8
Soybean	55.6	32.2	30.9
Red bean	55.6	32.2	30.9
Peanut	55.6	32.2	30.9
Goji berries	55.6	32.2	30.9
Yellow corn	55.6	32.2	30.9
Lotus seed	55.6	32.2	30.9
Big jujube	55.6	32.2	30.9
Olive oil	16.2	14.8	14.6
**Formula 2**			
Bitter gourd	36.6	43.1	43.1
Fibersol-2	2.44	2.87	2.87
Oligosaccharides	0.61	0.72	0.72
Isomaltose	1.02	1.20	1.20
**Formula 3**			
Fibersol-2	16.1	16.1	53.6
Oligosaccharides	4.02	4.02	13.4
Isomaltose	6.70	6.70	22.3

## Data Availability

The original sequencing data used during the current study are available in the NCBI SRA database with accession number SRP045211. The tables, figures and supplemental materials supporting the conclusions of the article are included within the article.

## Supplementary Materials

The following are available online at https://www.mdpi.com/article/10.3390/microorganisms9122493/s1, Table S1: Total flux intake, Table S2: Manually added flux for adlay, and Table S3: Manually added flux for other nutritions.
