# Supplementary material for: Metagenome-Scale Metabolic Network Suggests Folate Produced by Bifidobacterium Longum Might Contribute to High-Fiber-Diet-Induced Weight Loss in a Prader–Willi Syndrome Child
Source: Microorganisms. 2021 Dec 1;9(12):2493. doi: 10.3390/microorganisms9122493 (PMC8705902; doi:10.3390/microorganisms9122493)
Supplement: Supplementary file 1 [file microorganisms-09-02493-s001.zip › microorganisms-1459188-supplementary.pdf]

Table S1: Total flux intake

flux	D0, D15, D30 (mmol/day)	D45, D60 (mmol/day)	D75, D105 (mmol/day)
**EX_5mthf(e)**	3.47E-04	2.16E-04	2.09E-04
**EX_ala_D(e)**	2.33E+01	1.34E+01	1.29E+01
**EX_ala_L(e)**	2.36E+01	1.37E+01	1.31E+01
**EX_arach(e)**	2.15E-01	1.97E-01	1.94E-01
**EX_arg_L(e)**	3.72E+01	2.15E+01	2.07E+01
**EX_ascb_L(e)**	1.18E+00	7.90E-01	7.67E-01
**EX_asp_D(e)**	3.32E+01	1.92E+01	1.84E+01
**EX_asp_L(e)**	3.34E+01	1.93E+01	1.85E+01
**EX_avite1(e)**	1.86E-02	1.26E-02	1.22E-02
**EX_ca2(e)**	1.33E+01	8.17E+00	7.88E+00
**EX_caro(e)**	2.25E-04	1.30E-04	1.25E-04
**EX_CE2510(e)**	7.41E+00	4.34E+00	4.17E+00
**EX_chol(e)**	1.43E+00	8.27E-01	7.94E-01
**EX_cellul(e)**	5.93E-02	3.43E-02	3.29E-02
**EX_cu2(e)**	6.55E-02	3.86E-02	3.71E-02
**EX_cys_L(e)**	2.63E-02	1.52E-02	1.46E-02
**EX_dca(e)**	1.95E-02	1.13E-02	1.08E-02
**EX_ddca(e)**	2.23E-01	1.29E-01	1.24E-01
**EX_doco13ac(e)**	5.77E-02	3.33E-02	3.20E-02
**EX_docosac(e)**	6.14E-02	5.62E-02	5.55E-02
**EX_fe2(e)**	4.54E-01	2.67E-01	2.57E-01
**EX_fe3(e)**	2.47E-01	1.47E-01	1.41E-01
**EX_fol(e)**	4.43E-04	2.72E-04	2.62E-04
**EX_fru(e)**	6.67E+01	3.86E+01	3.70E+01
**EX_glc_D(e)**	6.03E+01	3.48E+01	3.35E+01
**EX_glu_L(e)**	1.08E+02	6.27E+01	6.02E+01
**EX_gly(e)**	5.57E+01	3.22E+01	3.09E+01
**EX_h2o(e)**	2.60E+04	1.61E+04	1.56E+04
**EX_hdca(e)**	3.86E+01	2.47E+01	2.39E+01
**EX_hdcea(e)**	1.42E+00	1.09E+00	1.07E+00
**EX_his_L(e)**	1.36E+01	7.87E+00	7.56E+00
**EX_hpdca(e)**	2.08E+00	1.21E+00	1.16E+00
**EX_ile_L(e)**	2.62E+01	1.51E+01	1.45E+01
**EX_k(e)**	1.17E+02	7.09E+01	6.84E+01
**EX_leu_L(e)**	4.72E+01	2.73E+01	2.62E+01
**EX_lgnc(e)**	1.76E+00	1.01E+00	9.74E-01
**EX_lnlc(e)**	6.63E+01	4.04E+01	3.89E+01
**EX_lys_L(e)**	2.72E+01	1.57E+01	1.51E+01
**EX_malt(e)**	2.76E-01	1.60E-01	1.53E-01

**EX_met_L(e)**	8.31E+00	4.80E+00	4.61E+00
**EX_mg2(e)**	4.52E+01	2.69E+01	2.59E+01
**EX_mn2(e)**	6.48E-01	3.76E-01	3.62E-01
**EX_na1(e)**	1.00E+01	5.90E+00	5.67E+00
**EX_nac(e)**	5.52E-02	3.29E-02	3.17E-02
**EX_ncam(e)**	5.52E-02	3.29E-02	3.17E-02
**EX_ocdca(e)**	7.26E+00	4.57E+00	4.42E+00
**EX_ocdcea(e)**	1.46E+02	9.80E+01	9.51E+01
**EX_octa(e)**	4.66E-02	2.69E-02	2.59E-02
**EX_phe_L(e)**	2.47E+01	1.43E+01	1.37E+01
**EX_phyQ(e)**	6.31E-05	4.37E-05	4.25E-05
**EX_pi(e)**	2.76E+01	1.62E+01	1.55E+01
**EX_pnto_R(e)**	2.88E-02	1.67E-02	1.60E-02
**EX_pro_D(e)**	1.90E+01	1.10E+01	1.06E+01
**EX_pro_L(e)**	1.92E+01	1.11E+01	1.07E+01
**EX_pydam(e)**	3.10E-03	2.14E-03	2.08E-03
**EX_pydx(e)**	3.14E-03	2.16E-03	2.11E-03
**EX_pydxn(e)**	3.10E-03	2.14E-03	2.08E-03
**EX_ribflv(e)**	2.88E-03	1.88E-03	1.82E-03
**EX_ser_L(e)**	3.95E+01	2.28E+01	2.19E+01
**EX_starch1200(e)**	7.06E-01	4.08E-01	3.92E-01
**EX_strch1(e)**	7.38E-01	4.27E-01	4.10E-01
**EX_strch2(e)**	2.64E+00	1.52E+00	1.46E+00
**EX_sucr(e)**	1.98E+01	1.14E+01	1.10E+01
**EX_thf(e)**	3.59E-04	2.23E-04	2.16E-04
**EX_thm(e)**	9.61E-03	5.70E-03	5.49E-03
**EX_thr_L(e)**	2.61E+01	1.51E+01	1.45E+01
**EX_trp_L(e)**	4.54E+00	2.62E+00	2.52E+00
**EX_ttdca(e)**	3.19E-01	1.84E-01	1.77E-01
**EX_tyr_L(e)**	1.48E+01	8.54E+00	8.20E+00
**EX_val_L(e)**	3.61E+01	2.09E+01	2.01E+01
**EX_zn2(e)**	3.37E-01	1.96E-01	1.88E-01
**EX_HC00229(e)**	2.26E+01	2.31E+01	6.88E+01
**EX_dextrin(e)**	1.87E+01	1.91E+01	5.70E+01
**EX_kesto(e)**	3.06E+00	3.13E+00	9.33E+00
**EX_kestopt(e)**	1.86E+00	1.91E+00	5.68E+00
**EX_kestottr(e)**	2.32E+00	2.37E+00	7.06E+00

Table S2: Manually added flux for adlay

metabolites in flux	adlay composition (g metabolites/100g adlay)	D0, D15, D30 (mmol/day)	D45, D60 (mmol/day)	D75, D105 (mmol/day)
**EX_etoh[e]**		0.00E+00	0.00E+00	0.00E+00
**EX_h2o[e]**	8.79E+00	1.09E+03	6.28E+02	6.03E+02
**EX_caro[e]**		0.00E+00	0.00E+00	0.00E+00
**EX_retinol[e]**		0.00E+00	0.00E+00	0.00E+00
**EX_thm[e]**	2.00E-05	1.68E-04	9.69E-05	9.31E-05
**EX_adpcbl[e]**		0.00E+00	0.00E+00	0.00E+00
**EX_ribflv[e]**	5.00E-05	2.96E-04	1.71E-04	1.64E-04
**EX_nac[e]**		0.00E+00	0.00E+00	0.00E+00
**EX_ncam[e]**		0.00E+00	0.00E+00	0.00E+00
**EX_pnto_R[e]**	5.00E-04	5.10E-03	2.95E-03	2.83E-03
**EX_pydam[e]**		0.00E+00	0.00E+00	0.00E+00
**EX_pydxn[e]**		0.00E+00	0.00E+00	0.00E+00
**EX_pydx[e]**		0.00E+00	0.00E+00	0.00E+00
**EX_btn[e]**		0.00E+00	0.00E+00	0.00E+00
**EX_10fthf[e]**		0.00E+00	0.00E+00	0.00E+00
**EX_5mthf[e]**		0.00E+00	0.00E+00	0.00E+00
**EX_thf[e]**		0.00E+00	0.00E+00	0.00E+00
**EX_ascb_L[e]**		0.00E+00	0.00E+00	0.00E+00
**EX_vitd3[e]**		0.00E+00	0.00E+00	0.00E+00
**EX_avite1[e]**	1.00E-04	5.17E-04	2.99E-04	2.87E-04
**EX_phyQ[e]**		0.00E+00	0.00E+00	0.00E+00
**EX_ca2[e]**	1.05E-02	5.85E-01	3.38E-01	3.25E-01
**EX_cl[e]**		0.00E+00	0.00E+00	0.00E+00
**EX_k[e]**	2.74E-01	1.56E+01	9.03E+00	8.67E+00
**EX_mg2[e]**	1.73E-01	1.58E+01	9.15E+00	8.78E+00
**EX_na1[e]**	9.00E-03	8.71E-01	5.03E-01	4.83E-01
**EX_pi[e]**	3.62E-01	8.40E+00	4.85E+00	4.66E+00
**EX_cu2[e]**	5.39E-04	1.89E-02	1.09E-02	1.05E-02
**EX_fe2[e]**	5.21E-03	2.08E-01	1.20E-01	1.15E-01
**EX_fe3[e]**		0.00E+00	0.00E+00	0.00E+00
**EX_mn2(e)**	2.79E-03	1.13E-01	6.53E-02	6.27E-02
**EX_zn2(e)**	4.14E-03	1.41E-01	8.15E-02	7.83E-02
**EX_mnl(e)**		0.00E+00	0.00E+00	0.00E+00
**EX_xylt[e]**		0.00E+00	0.00E+00	0.00E+00
**EX_lcts[e]**		0.00E+00	0.00E+00	0.00E+00
**EX_malt[e]**		0.00E+00	0.00E+00	0.00E+00
**EX_sucr[e]**		0.00E+00	0.00E+00	0.00E+00

**EX_fru[e]**		0.00E+00	0.00E+00	0.00E+00
**EX_gal[e]**		0.00E+00	0.00E+00	0.00E+00
**EX_cellul(e)**	2.16E+00	5.93E-02	3.43E-02	3.29E-02
**EX_ala_L[e]**	1.54E-02	3.83E-01	2.22E-01	2.13E-01
**EX_arg_L[e]**	6.20E-03	7.87E-02	4.55E-02	4.37E-02
**EX_asp_L[e]**	9.44E-03	1.59E-01	9.19E-02	8.83E-02
**EX_cys_L[e]**	1.43E-03	2.63E-02	1.52E-02	1.46E-02
**EX_glu_L[e]**	3.56E-02	5.42E-01	3.13E-01	3.01E-01
**EX_gly[e]**	3.53E-03	1.05E-01	6.05E-02	5.81E-02
**EX_urate[e]**	0.00E+00	0.00E+00	0.00E+00	0.00E+00
**EX_his_L[e]**	4.85E-03	6.96E-02	4.02E-02	3.86E-02
**EX_ile_L[e]**	5.90E-03	1.00E-01	5.79E-02	5.56E-02
**EX_leu_L[e]**	2.19E-02	3.71E-01	2.15E-01	2.06E-01
**EX_lys_L[e]**	2.54E-03	3.84E-02	2.22E-02	2.13E-02
**EX_met_L[e]**	1.30E-03	1.94E-02	1.12E-02	1.08E-02
**EX_phe_L[e]**	8.16E-03	1.10E-01	6.35E-02	6.10E-02
**EX_pro_L[e]**	1.09E-02	2.11E-01	1.22E-01	1.17E-01
**EX_ser_L[e]**	6.86E-03	1.45E-01	8.40E-02	8.06E-02
**EX_thr_L[e]**	4.48E-03	8.37E-02	4.84E-02	4.65E-02
**EX_trp_L[e]**	0.00E+00	0.00E+00	0.00E+00	0.00E+00
**EX_tyr_L[e]**	6.11E-03	7.50E-02	4.34E-02	4.17E-02
**EX_val_L[e]**	7.90E-03	1.50E-01	8.67E-02	8.33E-02
**EX_dca[e]**		0.00E+00	0.00E+00	0.00E+00
**EX_ddca[e]**		0.00E+00	0.00E+00	0.00E+00
**EX_ttdca[e]**	4.60E-03	4.50E-02	2.60E-02	2.50E-02
**EX_ttdcea[e]**		0.00E+00	0.00E+00	0.00E+00
**EX_ptdca[e]**		0.00E+00	0.00E+00	0.00E+00
**EX_hdca[e]**	1.12E+00	9.76E+00	5.64E+00	5.42E+00
**EX_hpdca[e]**	2.50E-01	2.06E+00	1.19E+00	1.15E+00
**EX_ocdca[e]**	3.60E-01	2.83E+00	1.63E+00	1.57E+00
**EX_ocdcea[e]**	4.91E+00	3.88E+01	2.24E+01	2.16E+01
**EX_lnlc[e]**		0.00E+00	0.00E+00	0.00E+00
**EX_lnlnca[e]**		0.00E+00	0.00E+00	0.00E+00
**EX_strdnc[e]**		0.00E+00	0.00E+00	0.00E+00
**EX_arach[e]**		0.00E+00	0.00E+00	0.00E+00
**EX_CE2510[e]**	8.40E-01	6.04E+00	3.49E+00	3.35E+00
**EX_CE4843[e]**		0.00E+00	0.00E+00	0.00E+00
**EX_arachd[e]**		0.00E+00	0.00E+00	0.00E+00
**EX_docosac[e]**		0.00E+00	0.00E+00	0.00E+00
**EX_doco13ac[e]**		0.00E+00	0.00E+00	0.00E+00
**EX_adrn[e]**		0.00E+00	0.00E+00	0.00E+00
**EX_clpnd[e]**		0.00E+00	0.00E+00	0.00E+00
**EX_crvnc[e]**		0.00E+00	0.00E+00	0.00E+00
**EX_but[e]**		0.00E+00	0.00E+00	0.00E+00

**EX_octa[e]**		0.00E+00	0.00E+00	0.00E+00
**EX_chsterol[e]**		0.00E+00	0.00E+00	0.00E+00
**EX_sbt_D[e]**		0.00E+00	0.00E+00	0.00E+00
**EX_glc_D[e]**		0.00E+00	0.00E+00	0.00E+00
**EX_hdcea[e]**	5.00E-02	4.39E-01	2.54E-01	2.44E-01
**EX_lgnc[e]**	2.90E-01	1.76E+00	1.01E+00	9.74E-01
**EX_fol[e]**	1.60E-05	8.08E-05	4.67E-05	4.49E-05
**EX_strch1[e]**		0.00E+00	0.00E+00	0.00E+00
**EX_i[e]**		0.00E+00	0.00E+00	0.00E+00
**EX_starch1200(e)**	6.12E+01	6.99E-01	4.04E-01	3.88E-01

Table S3: Manually added flux for other nutritions

other metabolites	D0, D15, D30 (mmol/day)	D45, D60 (mmol/day)	D75, D105 (mmol/day)
**isomaltose**	22.56	23.08	68.77
**dxtrin**	18.70	19.13	57.01
**kestose**	3.06	3.13	9.33
**kestopentaose**	1.86	1.91	5.68
**kestotetraose**	2.32	2.37	7.06
